# A rhesus macaque model of Asian-lineage Zika virus infection

**DOI:** 10.1038/ncomms12204

**Published:** 2016-06-28

**Authors:** Dawn M. Dudley, Matthew T. Aliota, Emma L. Mohr, Andrea M. Weiler, Gabrielle Lehrer-Brey, Kim L. Weisgrau, Mariel S. Mohns, Meghan E. Breitbach, Mustafa N. Rasheed, Christina M. Newman, Dane D. Gellerup, Louise H. Moncla, Jennifer Post, Nancy Schultz-Darken, Michele L. Schotzko, Jennifer M. Hayes, Josh A. Eudailey, M. Anthony Moody, Sallie R. Permar, Shelby L. O'Connor, Eva G. Rakasz, Heather A. Simmons, Saverio Capuano, Thaddeus G. Golos, Jorge E. Osorio, Thomas C. Friedrich, David H. O'Connor

**Affiliations:** 1 Department of Pathology and Laboratory Medicine, University of Wisconsin-Madison, Madison, Wisconsin 53705, USA; 2 Department of Pathobiological Sciences, University of Wisconsin-Madison, Madison, Wisconsin 53706, USA; 3 Department of Pediatrics, School of Medicine and Public Health, University of Wisconsin-Madison, Madison, Wisconsin 53705, USA; 4 Wisconsin National Primate Research Center, University of Wisconsin-Madison, Madison, Wisconsin 53715, USA; 5 Department of Pediatrics and Human Vaccine Institute, Duke University Medical Center, Durham, North Carolina 27710, USA; 6 Department of Comparative Biosciences and Obstetrics and Gynecology, University of Wisconsin-Madison, Madison, Wisconsin 53706, USA

## Abstract

Infection with Asian-lineage Zika virus (ZIKV) has been associated with Guillain–Barré syndrome and fetal abnormalities, but the underlying mechanisms remain poorly understood. Animal models of infection are thus urgently needed. Here we show that rhesus macaques are susceptible to infection by an Asian-lineage ZIKV closely related to strains currently circulating in the Americas. Following subcutaneous inoculation, ZIKV RNA is detected in plasma 1 day post infection (d.p.i.) in all animals (*N*=8, including 2 pregnant animals), and is also present in saliva, urine and cerebrospinal fluid. Non-pregnant and pregnant animals remain viremic for 21 days and for up to at least 57 days, respectively. Neutralizing antibodies are detected by 21 d.p.i. Rechallenge 10 weeks after the initial challenge results in no detectable virus replication, indicating protective immunity against homologous strains. Therefore, Asian-lineage ZIKV infection of rhesus macaques provides a relevant animal model for studying pathogenesis and evaluating potential interventions against human infection, including during pregnancy.

## Abstract (web summary)

Animal models of infection with Zika virus (ZIKV) are urgently needed for a better understanding of pathogenesis and for testing potential therapies. Here, the authors describe infection of rhesus macaques with an Asian-lineage ZIKV strain as a relevant animal model for studying ZIKV pathogenesis.

## Graphical abstract

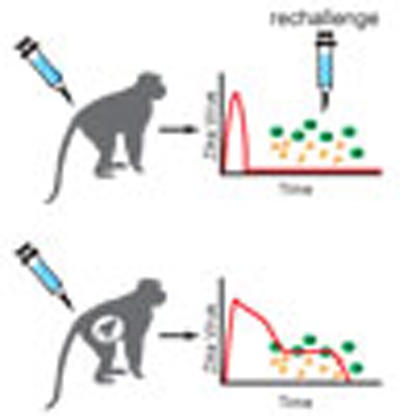

Zika virus (ZIKV) is a mosquito-borne flavivirus first identified in 1947 (ref. 1). Little was known about ZIKV when fetal abnormalities and Guillain–Barré syndrome were reported coincident with epidemic spread of Asian-lineage ZIKV in South America^2^,^3^,^4^. Animal models are essential for quickly understanding ZIKV transmission and pathogenesis, as well as for evaluating candidate vaccines and therapeutics. ZIKV infects immunocompromised mice^5^, providing evidence suggesting that ZIKV causes microcephaly by attacking neuronal progenitor cells and leads to intrauterine growth restriction^6^,^7^,^8^,^9^. However, mouse models do not mimic key attributes of human infection and fetal development such as infection in an immunocompetent state or the same type of remodelling of the spiral arteries and arterial vasodilation^10^. This remodelling of the arteries that occurs in humans and nonhuman primates, but not in mice, leads to high blood flow to the fetus, altering the potential transmission of viruses.

In contrast to mice, immunocompetent macaque monkeys are widely used in both infectious disease and obstetric research, with similar gestation and fetal development as humans. Here we describe infection of rhesus macaques with an Asian-lineage ZIKV strain.

## Results

### Cohort definitions

**Figure 1:**
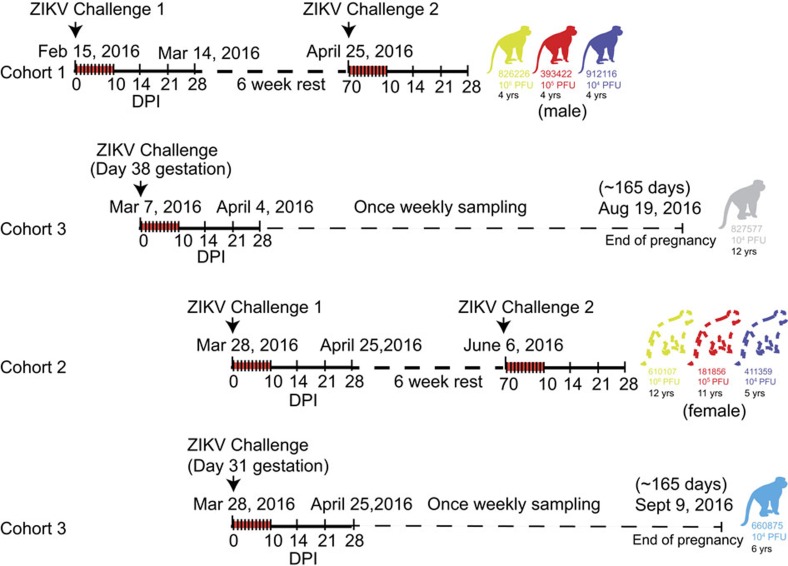
Schematic representation of the timeline of infection and sampling for each animal in the presented studies. Cohort 1 received the first ZIKV challenges and was then rested for 6 weeks before a rechallenge. For all studies, samples were collected daily for 10 days and then on 14, 21 and 28 d.p.i. as indicated by hashes in the timelines. Cohort 3 represents the two pregnant animals that were challenged on two different days. Both animals are currently in the once weekly sampling phase until the pregnancies come to term (∼165 gestational days). Cohort 2 was a repeat experiment of cohort 1 that allowed for additional experiments and sample collection (for example, serum plaque infectivity) that were not feasible when we initiated cohort 1 studies. These animals are currently in a 6-week rest period and will be rechallenged on 6 June 2016. Ages of all animals are indicated under each macaque identification number.

To determine whether a physiologically relevant dose and route of Asian-lineage ZIKV infects immunocompetent pregnant and non-pregnant macaques, we inoculated eight Indian-origin rhesus macaques (*Macaca mulatta*) subcutaneously with ZIKV derived from a French Polynesian virus isolate (Zika virus/H.sapiens-tc/FRA/2013/FrenchPolynesia-01_v1c1). The eight animals were divided into three cohorts as shown in Fig. 1. Cohort 1 (three male animals) received the first ZIKV challenges and were then rested for 6 weeks before a rechallenge. Cohort 2 (three female animals) was a repeat experiment of cohort 1 that allowed for additional experiments and sample collection (for example, serum infectivity) that were not feasible when we initiated cohort 1 studies. Animals in cohort 3 (two pregnant animals) were challenged on two different days. Both cohort 3 animals are currently in the once weekly sampling phase until the pregnancies come to term (∼165 gestational days).

### ZIKV titrations

**Figure 2:**
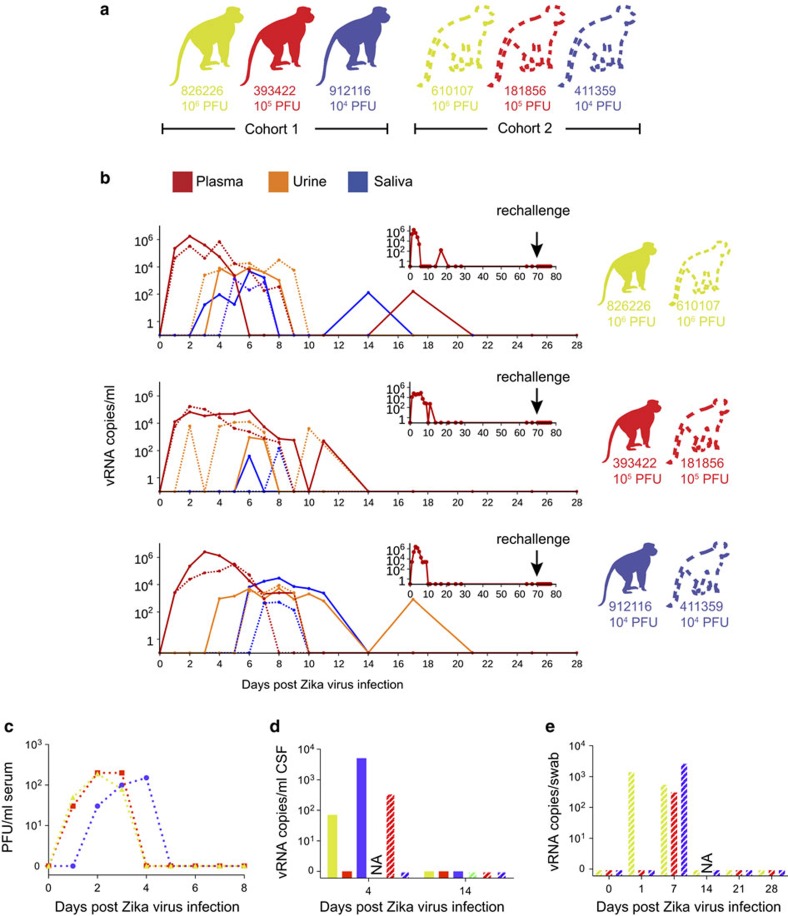
Animal cohort definitions and ZIKV viral load from rhesus macaque fluids. (**a**) Animals included in this study and the ZIKV doses used to infect them. Solid lines and bars throughout the figure represent cohort 1 animals by colour, while stripped bars and dotted lines represent cohort 2 animals by colour. (**b**) Viral RNA loads measured in plasma, urine and saliva for the two animals challenged with each dose of virus through 28 d.p.i. Cohort 1 animals are represented by a solid line, while cohort 2 animals are represented by a dotted line for each fluid. Inset: vRNA loads from cohort 1 animals measured before and after rechallenge with homotypic Zika virus as indicated by an arrow. (**c**) Number of plaque-forming units per ml of serum for cohort 2 animals. (**d**) Viral RNA load per ml of CSF collected on 4 and 14 d.p.i. (**e**) Viral RNA load per vaginal swab collected on 0, 7, 14, 21 and 28 d.p.i. NA, sample not available.

To define the minimal dosage necessary to establish infection, two macaques per group were infected with 1 × 10^6^, 1 × 10^5^ or 1 × 10^4^ plaque-forming unit (p.f.u.) ZIKV (cohorts 1 and 2; Figs 1 and 2a). This dose range of inocula is based on the previous work in related flaviviruses such as West Nile virus and dengue virus, where it is estimated that mosquitoes deliver 1 × 10^4^ to 1 × 10^6^ p.f.u. of virus^11^,^12^. This is also the range found in mosquito saliva in a recent publication specifically evaluating Brazilian Zika virus^13^.

### Clinical evaluation of ZIKV-infected rhesus macaques

To detect signs of morbidity, animals were evaluated daily for evidence of disease, injury or psychological abnormalities (for example, inappetence, dehydration, diarrhoea, depression, inactivity, trauma, self-injurious or stereotypical repetitive behaviours (for example, pacing) often seen in captive animals). Five of six animals exhibited mild-to-moderate inappetence, which resulted in mild weight loss in four animals. Two animals (912116 and 393422) also developed a very mild rash around the inoculation site at 1 day post infection (d.p.i.) that persisted for 4–5 days. No other abnormal clinical signs were noted (for example, increased body temperature, joint pain, lymphadenopathy and lethargy).

Daily complete blood counts (CBCs) were evaluated for all six non-pregnant animals for 10 d.p.i. and then every 3–7 days thereafter and serum chemistry analyses were performed intermittently post infection as per the protocol (Supplementary Fig. 1). Reference intervals (RIs) developed for the Wisconsin National Primate Research Center (WNPRC) colony for species, gender and age were used to evaluate results. All six animals developed elevated serum creatine kinase (CK), which peaked by 5 d.p.i. (Supplementary Fig. 1d). Increases in serum CK are strongly associated with muscle damage and myositis (skeletal, smooth and cardiac), but can also be caused by repeated sedation, haemolysis and endocrine abnormalities^14^,^15^. Predictable increases in CK have been noted in nonhuman primates undergoing repeated sedation and venipuncture^15^. However, CK levels did not remain elevated during the 10-day period of daily sedation and blood collection, suggesting other causes for the noted increase in values. Future studies are planned to determine whether CK increases may be due to viral myositis. Alanine aminotransferase values in three of six animals exceeded the maximum WNPRC RI (Supplementary Fig. 1c) and the expected increase associated with repeated ketamine sedation^15^. Although aspartate aminotransferase values exceeded upper RI values for all six non-pregnant animals after infection, they did not increase above the expected levels previously associated with repeated ketamine sedation described by Lugo-Roman *et al*.^15^ (Supplementary Fig. 1b).

All the animals displayed decreased total white blood cell (WBC) numbers following infection, but only one animal fell below the RI with a value of 2.88 ths μl^−1^ (3.70–15.70). WBC numbers rebounded almost completely to pre-infection levels ∼10 d.p.i. in all six animals (Supplementary Fig. 1e). Both animals that received the highest dose of inoculum developed persistent mature neutrophilia ∼7–14 d.p.i. that lasted through 28 d.p.i. Five of six non-pregnant animals had mild regenerative anaemia characterized by varying degrees of polychromasia and anisocytosis, but whether this was secondary to the viral infection or simply a result of frequent blood collections could not be determined. Platelet values for all six animals remained within RI. Both cellular dyscrasias and elevated transaminases have been described in human ZIKV case reports; myositis has not been reported^16^,^17^.

### ZIKV detection in blood

Blood was sampled daily for 10–11 d.p.i. and every 3–7 days thereafter. Viral RNA (vRNA) was quantified by quantitative PCR with reverse transcription (qRT–PCR) from plasma^18^ and was detected in all six animals at 1 d.p.i. (Fig. 2b). Peak plasma viremia occurred between 2 and 6 d.p.i., and ranged from 8.2 × 10^4^ to 2.5 × 10^6^ vRNA copies per ml. Infectious titres, measured from serum in cohort 2 animals, were 500–1,000-fold less than copies of vRNA detected from plasma at the same time points (Fig. 2c). Copies of vRNA detected in the serum and plasma were very similar (Supplementary Fig. 2). The estimated doubling time for plasma viremia averaged 7.7 h (range=4.8–10.2 h) and was independent of the infecting dose and sex of the macaque. By 10 d.p.i., plasma viral loads were undetectable (<100 vRNA copies per ml) in all six animals, although intermittent low-level detection (<550 vRNA copies per ml) continued sporadically through 17 d.p.i. (Fig. 2b). Thereafter, vRNA remained undetectable in all fluids throughout follow-up (longest follow-up 70 d.p.i.; Fig. 2b, insets).

### ZIKV detection in other body fluids

We also measured ZIKV vRNA by qRT–PCR in other body fluids including urine, saliva, cerebrospinal fluid (CSF) and vaginal fluid. Viruria was detected starting at 2–5 d.p.i. and as late as 17 d.p.i., in urine passively collected from cage pans (Fig. 2b). Despite possible degradation of virus between the time of urination, and sample collection and processing, 1 × 10^3^–1 × 10^4^ vRNA copies per ml urine was detected at multiple time points. Virus was also detected in oral swabs collected from all six animals, peaking at over 1 × 10^3^ vRNA copies per sample in three of six animals (Fig. 2b). Notably, as with urine, the kinetics of virus detection in saliva occurred after peak plasma viremia. Cisterna magna punctures were performed at 4 and 14 d.p.i. to quantify vRNA in CSF; vRNA was detectable at 4 d.p.i. in three out of five animals from which CSF could be obtained (Fig. 2d). Vaginal swabs collected from the three female animals in cohort 2 had detectable vRNA starting at 1 and/or 7 d.p.i., but were undetectable at 14, 21 and 28 d.p.i. (Fig. 2e).

### Rhesus macaque innate and adaptive immune responses to ZIKV

**Figure 3:**
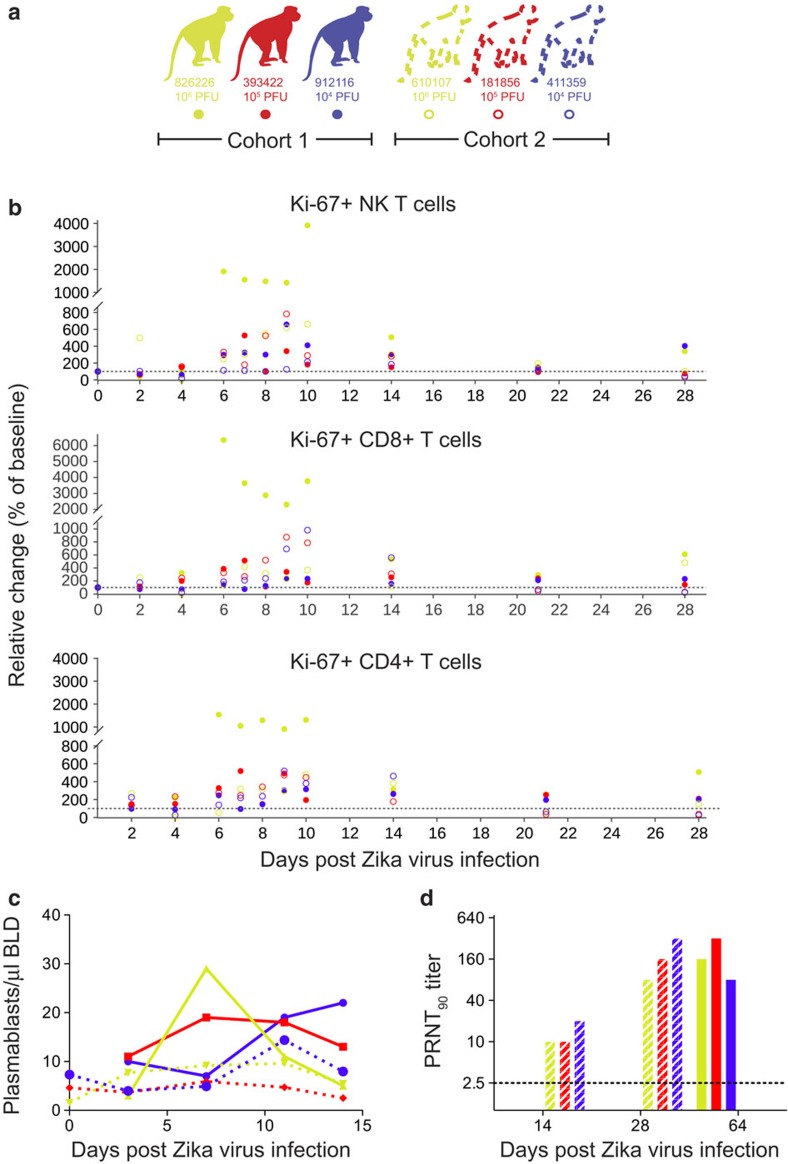
Immune cell expansion and neutralizing antibody titres following ZIKV infection. (**a**) Solid dots, lines and bars with corresponding colour represent cohort 1 animals and open circles, dotted lines or stripped bars represent cohort 2 animals throughout the figure. (**b**) Expansion of Ki-67^+^ (activated) NK cells, CD8^+^ T cells and CD4^+^ T cells were measured daily for 10 days and then on days 14, 21 and 28 post infection. Absolute numbers of activated cells per μl of blood are presented relative to the baseline value set to 100%. (**c**) Total number of plasmablast cells found in PBMCs collected at 0 (cohort 2 only), 3, 7, 11 and 14 d.p.i. for each animal. (**d**) PRNT_90_ titres for cohorts 1 and 2. The dotted line indicates the first dilution of serum tested.

We next characterized the immune response to infection by staining peripheral blood mononuclear cells (PBMCs) for multiple lineage and activation markers. Proliferating (Ki-67^+^) natural killer (NK) cells, CD8^+^ T cells and CD4^+^ T cells expanded above baseline levels by 6 d.p.i. (Fig. 3a,b). NK and CD8^+^ T-cell expansion increased as plasma vRNA loads decreased starting at 6 d.p.i. We also enumerated circulating plasmablasts, defined as CD3^−^/20^−^/14^−^/16^−^/11c^−^/123^−^ and CD80^+^/HLA-DR^+^ cells, on 0, 3, 7, 11 and 14 d.p.i. (Fig. 3c)^19^. The peak plasmablast expansion occurred between 7 and 10 d.p.i. in five out of six animals. Serum neutralizing antibody (nAb) responses were also measured by plaque reduction neutralization tests (PRNT_90_). All animals exhibited high nAb titres as early as 14 d.p.i. (Fig. 3d), the earliest time point tested. Cohort 1 animals were tested at 64 d.p.i. and cohort 2 animals were tested at 14 and 28 d.p.i. Together, these data suggest that peak activation of the adaptive immune response and antibody production occur 5–7 d.p.i. and may both be important to control viral replication as evidenced by reducing vRNA loads in the plasma at these time points.

To determine whether the activation of T cells correlated with the appearance of ZIKV-specific responses, we performed interferon-gamma (IFNγ) enzyme-linked immunosorbent spot (ELISPOT) on PBMCs collected at 4, 10 and 14 d.p.i. for animals in cohort 2. Cells were stimulated with pools of 15-mer peptides collectively representing the amino-acid sequence of the Asian-lineage NS5 protein (GenBank: KU321639). We detected specific IFNγ secretion in response to 12 of 16 peptide pools in at least one animal (Supplementary Fig. 3). Overall, this data support that there are ZIKV-specific T-cell responses in all animals tested.

### Protection from rechallenge with ZIKV

To determine whether the immune responses that we detected following primary challenge were protective against homotypic rechallenge, we rechallenged the three animals in cohort 1 10 weeks after primary infection with 1 × 10^4^ p.f.u. of the Zika virus/H.sapiens-tc/FRA/2013/FrenchPolynesia-01_v1c1 strain (Figs 1 and 2b, inset). Plasma, urine and saliva vRNA loads remain negative to at least 9 d.p.i. (as of 5 April 2016), indicating complete protection against ZIKV re-infection.

### ZIKV in pregnant macaques

**Figure 4:**
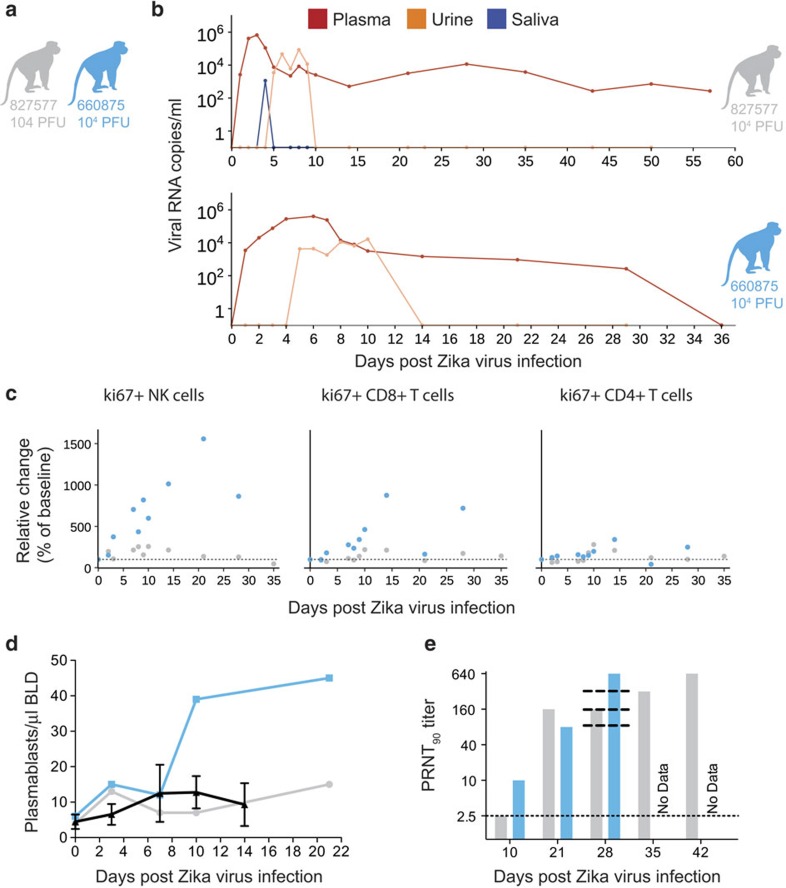
Characterization of Zika virus infection in animals infected during the first trimester of pregnancy. (**a**) Schematic of animals presented in this figure as cohort 3. Dots, lines and bars representing each animal match in colour throughout the figure. (**b**) vRNA copies of the plasma, urine and saliva from each pregnant animal. Oral swabs could not be obtained from 660875. (**c**) Absolute numbers of Ki-67^+^ NK, CD8^+^ T-cell and CD4^+^ T-cell populations presented as a percentage relative to baseline (× 100) over time in each animal. (**d**) Plasmablast expansion over time from each pregnant animal. The average plasmablast expansion of cohorts 1 and 2 animals infected with the 10^4^ p.f.u. is presented by the black line. Error bars represent s.d. (**e**) PRNT_90_ titres over time for each animal. Lines representing the titres from cohort 2 animals are overlaid at 28 d.p.i. for reference (top to bottom: 610107, 181856 and 411359).

We also challenged two time-mated rhesus macaques at approximately gestation day 31 and 38 (mid-first trimester) with 1 × 10^4^ p.f.u. of ZIKV (cohort 3; see Figs 1 and 4a). Both animals were viremic by 1 d.p.i. and exhibited peak plasma viral loads of >4 × 10^5^ vRNA copies per ml by 3 or 6 d.p.i. (Fig. 4b). Infectious virus was also quantified by plaque assay from the serum of 660875 (Supplementary Fig. 4). In contrast to their non-pregnant counterparts, both animals maintained persistent plasma viremia (vRNA copies per ml) to 57+ and 29 d.p.i. (Fig. 4b). This is similar to a case previously described by Driggers *et al*.^3^, where a pregnant mother had persistent ZIKV vRNA detected from 35 to 70 d.p.i. that did not resolve until termination of pregnancy; the fetus was found to have 2 × 10^8^ copies per ml of virus in brain tissue and it is speculated that the fetus may have been the source of the prolonged maternal plasma viremia. We will continue to monitor these pregnant animals for vRNA in the blood and amniotic fluid, and will determine the infection status of the fetus on termination of the pregnancy either at full term or earlier if necessary for the health and safety of the mother. Amniocentesis using ultrasound guidance was performed at 43 d.p.i. for animal 827577 and 36 d.p.i. for animal 660875, and both were negative for ZIKV RNA.

Both animals generated similar activation of NK, CD8^+^ T-cell and CD4^+^ T-cell responses above baseline as non-pregnant animals (Fig. 4c). Expansion of plasmablast cells was also observed by 10–21 d.p.i. with one animal expanding more than, and one animal expanding less than, the average non-pregnant animal (Fig. 4d). nAbs were detected by 21 d.p.i. for 827577 and 10 d.p.i. for 660875 and were similar to the cohort 2 (non-pregnant) animals at 28 d.p.i. (Fig. 4e). One pregnant animal exhibited an increase in CK above the RI and above levels expected with repeated ketamine sedation and blood collection. The same pregnant animal also developed persistent regenerative anaemia characterized by circulating nucleated erythrocytes.

## Discussion

Altogether, our study shows the persistence of ZIKV RNA in the plasma of rhesus macaques for ∼10 days, similar to other vector-borne flaviviruses that cause acute, typically self-limiting infections in humans. This work also shows that ZIKV infection elicits a robust immune response including ZIKV-specific T-cell response and nAb responses that confer protection against reinfection. However, the prolonged detection of vRNA in urine and saliva after apparent clearance from the blood, detection of virus in the CSF and occasional plasma ‘blips' after initial clearance suggest that ZIKV may persist longer, at low levels, in certain tissues. Future work in rhesus macaques will seek to determine whether and where these reservoirs may exist, and whether they seed virus into fluids that might allow for human-to-human transmission. Last, persistence of plasma viremia beyond 10 days was detected in both animals we infected during the first trimester of pregnancy. Future work will determine the source of that virus, as these pregnancies come to full term.

Our study establishes immunocompetent rhesus macaques infected with physiologically relevant ZIKV, as a relevant translational model for infection and pathogenesis. The large immunological toolset available for rhesus macaques will enable investigations of immunity and potential vaccines. Pregnancy, the maternal–fetal interface and fetal development have been described in detail in rhesus macaques, so this model will also enable assessments of the impact of maternal ZIKV infection on the developing fetus. We have established persistent viremia in pregnant macaques, despite activation of NK cells and T cells as well as development of a nAb response. We continue to follow these pregnant animals and will establish whether fetal infection and/or abnormalities have occurred through serial ultrasound assessments of the fetus and placenta as well as tissue analysis at pregnancy termination. Updates to all experiments are available in real time at the Zika Open-Research Portal (https://zika.labkey.com).

## Methods

### Study design

This was a proof-of-concept study designed to establish the infectivity and viral dynamics of Asian-lineage ZIKV. Because nothing is known about ZIKV dosing in macaques, one male and one female rhesus macaque of Indian ancestry were each challenged with the following ZIKV doses: 1 × 10^6^, 1 × 10^5^ and 1 × 10^4^ p.f.u. ZIKV. Two pregnant macaques at 31 and 38 days of gestation were infected with 1x10^4^ p.f.u. ZIKV. We selected two animals per inoculum dose and two pregnant animals as a minimum number of animals for this pilot study to provide proof of concept and design larger studies necessary to place statistical significance on the findings. All macaques utilized in the study were free of Macacine herpesvirus 1, simian retrovirus type D (SRV), simian T-lymphotropic virus type 1 (STLV) and simian immunodeficiency virus as part of the specific pathogen-free colony at WNPRC. All animals admitted into the specific pathogen-free colony at WNPRC were screened for each of these viruses quarterly for 1 year with serology test and additionally by PCR for simian retrovirus type D and simian T-lymphotropic virus type 1. Only animals with negative tests remained in the colony and all animals and their offspring are tested by serology and PCR yearly for these pathogens.

### Care and use of macaques

All macaque monkeys used in this study were cared for by the staff at the WNPRC in accordance with the regulations and guidelines outlined in the Animal Welfare Act and the Guide for the Care and Use of Laboratory Animals and the recommendations of the Weatherall report (https://royalsociety.org/topics-policy/publications/2006/weatherall-report/). This study was approved by the University of Wisconsin-Madison Graduate School Institutional Animal Care and Use Committee (Animal Care and Use Protocol Number G005401). For all procedures (that is, physical examinations, virus inoculations, ultrasound examinations, blood and swab collection), animals were anaesthetised with an intramuscular dose of ketamine (10 ml kg^−1^). Blood samples were obtained using a vacutainer system or needle and syringe from the femoral or saphenous vein.

### Inoculations

ZIKV strain H/PF/2013 (GenBank: KJ776791), originally isolated from a 51-year-old female in France returning from French Polynesia with a single round of amplification on Vero cells, was obtained from Xavier de Lamballerie (European Virus Archive, Marseille, France). We deep sequenced the challenge stock to verify the expected origin (see details in a section below). The ZIKV challenge stock consensus sequence matched the GenBank sequence (KJ776791) of the parental virus, but there were eight sites where between 5 and 40% of sequences contained variants that appear to be authentic (six out of eight were non-synonymous changes; Supplementary Table 1).

Virus stocks were prepared by inoculation onto a confluent monolayer of C6/36 mosquito cells. These cell lines were obtained from American Type Culture Collection (CRL-1660), were not further authenticated and were not specifically tested for mycoplasma. A single harvest of virus with a titre of 1.26 × 10^6^ p.f.u. ml^−1^ (equivalent to 1.43 × 10^9^ vRNA copies per ml) was used for all eight challenges. The stock was thawed, diluted in PBS to the appropriate concentration for each challenge and loaded into a 1-ml syringe that was kept on ice until challenge. Animals were anaesthetised as described above, and 1 ml of inocula was administered subcutaneously over the cranial dorsum. Post inoculation, animals were closely monitored by veterinary and animal care staff for adverse reactions and signs of disease.

### vRNA isolation from plasma

Fresh plasma and PBMC were isolated from EDTA-treated whole blood by Ficoll density centrifugation at 1,860 r.c.f. for 30 min. The plasma layer was collected and centrifuged for an additional 8 min at 670 r.c.f. to remove residual cells. RNA was extracted from 300 μl of plasma using the Viral Total Nucleic Acid Purification kit (Promega, Madison, WI, USA) on a Maxwell 16 MDx instrument. The RNA was then quantified by quantitative RT–PCR as described in a section below.

### vRNA isolation from urine

Urine was collected from a pan beneath the animal's cage. Urine was centrifuged for 5 min at 500 r.c.f. to remove cells and other debris. RNA was isolated from 300 μl of urine using the Viral Total Nucleic Acid Purification kit (Promega) on a Maxwell 16 MDx instrument.

### vRNA isolation from oral swabs

Oral swab samples were collected from infected animals while anaesthetised by gently running a sterile swab under the animal's tongue. Swabs were placed immediately into either RNAlater or viral transport medium (tissue culture medium 199 supplemented with 0.5% fetal bovine serum (FBS) and 1% antibiotic/antimycotic) for 60–90 min. Samples were vortexed vigorously, then centrifuged for 5 min at 500 r.c.f. before removing the swabs. Samples were stored at either −20 °C (RNAlater samples) or −80 °C (viral transport medium) until processing. Before extraction, virus was pelleted by centrifugation for 1 h at 4 °C at 14,000 r.p.m. Supernatant was removed, leaving the virus in 200 μl of media. vRNA was extracted from these samples using the Qiamp MinElute Virus Spin kit (Qiagen, Germantown, MD, USA) with all optional washes. Viral load data from oral swabs are expressed as vRNA copies per ml eluate.

### Quantitative reverse transcription–PCR

vRNA isolated from plasma, urine or oral swabs was quantified by qRT–PCR using the primers and probe designed by Lanciotti *et al*.^18^. The RT–PCR was performed using the SuperScript III Platinum one-step quantitative RT–PCR system (Invitrogen, Carlsbad, CA, USA) on the LightCycler 480 instrument (Roche Diagnostics, Indianapolis, IN, USA). Primers and probe were used at final concentrations of 600 and 100 nM, respectively, along with 150 ng random primers (Promega). Cycling conditions were as follows: 37 °C for 15 min, 50 °C for 30 min and 95 °C for 2 min, followed by 50 cycles of 95 °C for 15 s and 60 °C for 1 min. Virus concentration was determined by interpolation onto an internal standard curve composed of seven 10-fold serial dilutions of a synthetic ZIKV RNA fragment based on the Asian-lineage (ZIKV strain H/PF/2013).

### Viral quantification by plaque assay

Titrations for replication competent virus quantification of the challenge stock as well as from serum collected at multiple time points from animals in cohort 2 were completed by plaque assay on Vero cell cultures. Vero cells were obtained from American Type Culture Collection (CCL-81), were not further authenticated and were not specifically tested for mycoplasma. Duplicate wells were infected with 0.1 ml of aliquots from serial 10-fold dilutions in growth media and virus was adsorbed for 1 h. Following incubation, the inoculum was removed, and monolayers were overlaid with 3 ml containing a 1:1 mixture of 1.2% oxoid agar and 2 × DMEM (Gibco, Carlsbad, CA, USA) with 10% (vol/vol) FBS and 2% (vol/vol) penicillin/streptomycin. Cells were incubated at 37 °C in 5% CO_2_ for 4 days for plaque development. Cell monolayers then were stained with 3 ml of overlay containing a 1:1 mixture of 1.2% oxoid agar and 2 × DMEM with 2% (vol/vol) FBS, 2% (vol/vol) penicillin/streptomycin and 0.33% neutral red (Gibco). Cells were incubated overnight at 37 °C and plaques were counted. Titres of virus detected from the serum of cohort 2 animals were compared with plasma and serum viral load assays. For both the challenge stock and the virus isolated from macaque serum, the level of infectious virus detected by plaque assay was ∼500–1,000-fold less than the number of vRNA particles detected by qRT–PCR in either the plasma or serum. This was true throughout the duration of viremia, where plaque assay titres were detectable.

### Plaque reduction neutralization test

Macaque serum samples were screened for ZIKV nAb utilizing a PRNT. End point titrations of reactive sera, utilizing a 90% cutoff (PRNT_90_), were performed as described^20^ against ZIKV strain H/PF/2013. Briefly, plaque assays were set up as described above with the challenge stock of ZIKV in the presence of different dilutions of serum from the rhesus macaque.

### Immunophenotyping

The amount of activated/proliferating NK cells was quantified using a modified version of our protocol detailed step by step in OMIP-028 (ref. 21). Briefly, 0.1 ml of EDTA-anticoagulated whole-blood samples were incubated for 15 min at room temperature in the presence of a mastermix of antibodies against CD45 (clone D058-1283, Brilliant Violet 786 conjugate, 2.5 μl), CD3 (clone SP34-2 Alexa Fluor 700 conjugate, 5 μl), CD8 (clone SK2, Brilliant Violet 510, 2.5 μl), NKG2A/C (clone Z199, PE-Cy7 conjugate, 5 μl), CD16 (clone 3G8, Pacific Blue conjugate, 5 μl), CD69 (clone TP1.55.3, ECD conjugate, 3 μl), HLA-DR (clone 1D11, Brilliant Violet 650 conjugate, 1 μl), CD4 (clone SK3, Brilliant Violet 711 conjugate, 5 μl), CD28 (clone CD28.2, PE conjugate, 5 μl) and CD95 (clone DX2, PE-Cy5 conjugate, 10 μl) antigens. All antibodies were obtained from BD BioSciences, San Jose, CA, USA, except the NKG2A/C-specific antibody, which was purchased from Beckman Coulter, and the CCR7 antibody that was purchased from R&D Systems. Red blood cells were lysed using BD Pharm Lyse, after which they were washed twice in media and fixed with 0.125 ml of 2% paraformaldehyde for 15 min. After an additional wash the cells were permeabilized using Life Technology's Bulk Permeabilization Reagent. The cells were stained for 15 min with Ki-67 (clone B56, Alexa Fluor 647 conjugate) while the permeabilizer was present. The cells were then washed twice in media and resuspended in 0.125 ml of 2% paraformaldehyde until they were run on a BD LSRII Flow Cytometer. Flow data were analysed using Flowjo version 9.8.2.

### IFNγ ELISPOT assay

PBMCs were isolated from EDTA-treated whole blood using Ficoll-Paque Plus (GE Health Sciences) density centrifugation. ELISPOT assays were conducted according to the manufacturer's protocol. Briefly, 1 × 10^5^ cells in 100 μl of R10 medium were added to pre-coated monkey IFNγ ELISpot-PLUS plates (Mabtech Inc., Mariemont, OH, USA) with peptide at a final concentration of 1 μM. Full proteome peptides derived from the ZIKV *NS5* sequence (GenBank: KU321639.1) used in this study were synthesized by GenScript (Piscataway, NJ, USA). Pools were created using 10 overlapping 15-mer peptides, each at a working concentration of 1 mM. Concanavalin A (10 μM) was used as a positive control. Assays of all samples were repeated in duplicate or triplicate. Cells alone in the absence of stimulant were used as a negative control. Wells were imaged by using an AID ELISPOT reader, and spots were counted using an automated program with parameters including size, intensity and gradient. The limit of detection was set at 100 spot-forming cells per million PBMCs.

### Plasmablast detection

PBMCs isolated from three ZIKV-infected rhesus monkeys at 3, 7, 11 and 14 d.p.i. were stained with the following panel of fluorescently labelled Abs specific for the following surface markers: CD20 FITC (L27), CD80 PE (L307.4), CD123 PE-Cy7 (7G3), CD3 APC-Cy7 (SP34-2), IgG BV605(G18-145; all from BD Biosciences), CD14 AF700 (M5E2), CD11c BV421 (3.9), CD16 BV570 (3G8), CD27 BV650 (O323; all from BioLegend, San Diego, CA, USA), IgD AF647 (polyclonal; Southern Biotech, Birmingham, AL, USA) and HLA-DR PE-TxRed (TÜ36; Invitrogen). LIVE/DEAD Fixable Aqua Dead Cell Stain kit (Invitrogen) was used to discriminate live cells. Briefly, cells were resuspended in 1 × PBS/1%BSA and stained with the full panel of surface Abs for 30 min in the dark at 4 °C, washed once with 1 × PBS, stained for 30 min with the LIVE/DEAD Fixable Aqua Dead Cell Stain kit in the dark at 4 °C, washed once with 1 × PBS, washed again with 1 × PBS/1%BSA and resuspended in 2% paraformaldehyde Solution. Stained PBMCs were acquired on a LSRII Flow Analyser (BD Biosciences) and the data were analysed using FlowJo software v9.7.6 (TreeStar, Ashland, OR, USA). Plasmablasts were defined similarly to the method previously described^19^ excluding lineage cells (CD14^+^, CD16^+^, CD3^+^, CD20^+^, CD11c^+^ and CD123^+^), and selecting CD80^+^ and HLA-DR^+^ cells (known to be expressed on rhesus plasmablasts and their human counterpart^22^).

### Estimation of plasma viremia doubling time

The doubling time of plasma viremia was estimated in R version 3.2.3 (The R Foundation for Statistical Computing; http://www.R-project.org). For each animal, the slope of the linear portion of the line (between 1 and 2 d.p.i. for the animals treated with 1 × 10^6^ and 1 × 10^5^ p.f.u., and between 1, 2 and 3 d.p.i. for the animal treated with 1 × 10^4^ p.f.u.) was generated by plotting the log of the plasma viral loads. The linear portion represents the exponential growth phase and has been used to estimate doubling time in other systems^23^. The slopes were then used in the equation: log(2)/slope. Each result was then multiplied by 24 h to produce a simple estimate of doubling time in hours.

### CBC and blood chemistry panels

CBCs were performed on EDTA-anticoagulated whole-blood samples on a Sysmex XS-1000i automated haematology analyser (Sysmex Corporation, Kobe, Japan). Blood smears were prepared and stained with Wright-Giemsa stain (Wescor Aerospray Hematology Slide Stainer; Wescor Inc, Logan, UT, USA). Manual slide evaluations were performed on samples as appropriate when laboratory-defined criteria were met (including the presence of increased total WBC counts, increased monocyte, eosinophil and basophil percentages, decreased haemoglobin, haematocrit and platelet values, and unreported automated differential values). Individuals performing manual slide evaluations screened both WBCs and red blood cells for cellular maturity, toxic change and morphologic abnormalities.

Whole blood was collected into serum separator tubes (Becton, Dickinson and Company, Franklin Lakes, NJ, USA) for blood chemistry analysis and processed as per the manufacturer's instructions. Blood chemistry panels were performed on the serum using a Cobas 6000 analyser (Roche Diagnostics, Risch-Rotkreuz, Switzerland). Results from CBC and blood chemistry panels were reported with species, age and sex-specific reference ranges.

### ZIKV deep sequencing of the challenge stock

A vial of the same ZIKV strain H/PF/2013 virus stock that infected macaques was deep sequenced by preparing libraries of fragmented double-stranded complementary DNA using methods similar to those previously described^24^. Briefly, the sample was centrifuged at 5,000 r.c.f. for 5 min. The supernatant was then filtered through a 0.45-μm filter. The Qiagen QiAmp Minelute viral RNA isolation kit (omitting carrier RNA) was used to isolate vRNA. The eluted RNA was then treated with DNAse I. Double-stranded DNA was prepared with the Superscript double-stranded complementary DNA synthesis kit (Invitrogen) and priming with random hexamers. Agencourt Ampure XP beads were used to purify double-stranded DNA. The purified DNA was fragmented with the Nextera XT kit (Illumina), tagged with Illumina-compatible primers and then purified with Agencourt Ampure XP beads. Purified libraries were then sequenced with 2 × 300 bp kits on an Illumina MiSeq. Of note, challenge stock viral loads were 1.43 × 10^9^ vRNA copies per ml. This results in an input of 7.15 × 10^8^ RNA copies into the sequencing reactions. This far exceeds the average depth of coverage of 11,877 (±4,658) sequences per nucleotide site indicating little resampling effects in our data analysis.

### Data availability

Primary data that support the findings of this study are available at the Zika Open-Research Portal (https://zika.labkey.com). ZIKV sequence data have been deposited in the Sequence Read Archive with the accession code SRP072852. The authors declare that all other data supporting the findings of this study are available within the article and its Supplementary Information files, or from the corresponding author on request.

## Additional information

**How to cite this article:** Dudley, D. M. *et al*. A rhesus macaque model of Asian-lineage Zika virus infection. *Nat. Commun.* 7:12204 doi: 10.1038/ncomms12204 (2016).

## Supplementary Material

Supplementary Information Supplementary Figures 1-4 and Supplementary Table 1

Peer Review File
