# Supplementary material for: A rhesus macaque model of Asian-lineage Zika virus infection
Source: Nat Commun. 2016 Jun 28;7:12204. doi: 10.1038/ncomms12204 (PMC4931337; doi:10.1038/ncomms12204)
Supplement: Supplementary Information — Supplementary Figures 1-4 and Supplementary Table 1 [file ncomms12204-s1.pdf]

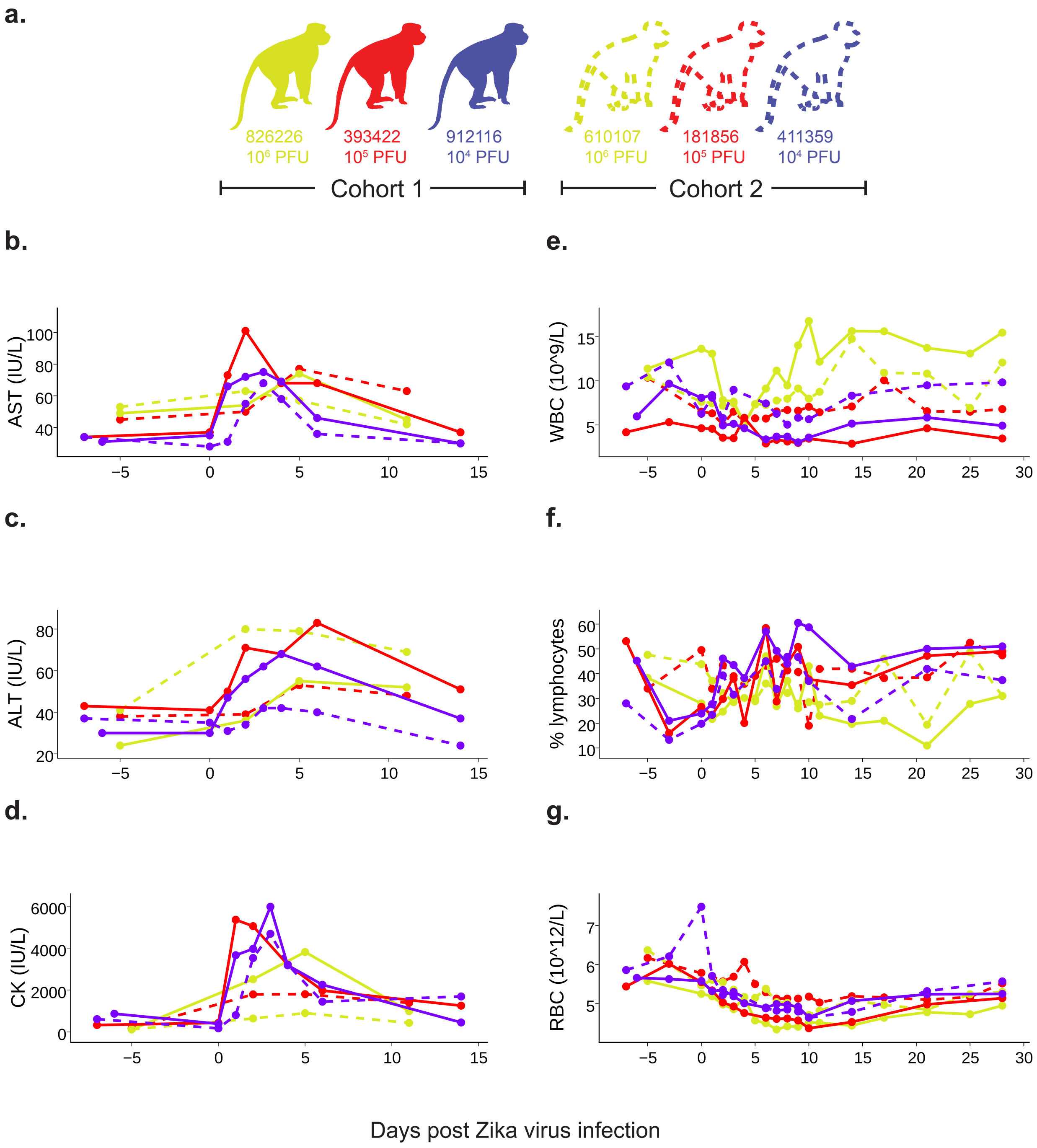

Supplementary Figure 1| Complete blood counts and serum chemistries for macaques infected with ZIKV. a. Animals were infected with different doses of ZIKV. Cohort 1 animals are represented by solid lines and cohort 2 animals are represented by dotted lines. All non-pregnant animals had serum chemistry analysis performed at -7, 0, 1, 2, 3, 4, 6, and 14 dpi or at -6, 2, 5 and 11 dpi. **b.** AST blood chemistries **c.** ALT serum chemistries. **d.** CK serum chemistries. Complete blood counts were measured prior to infection, daily for 10-11 days after infection and then every 3-7 days until 28 dpi. **e.** white blood cell counts. **f.** % lymphocytes. **g.** red blood cell counts.

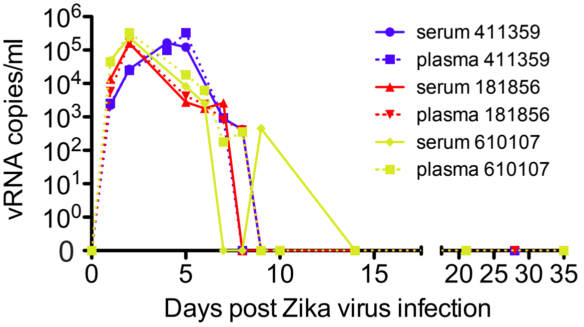

Supplementary Figure 2| qRT-PCR detection of ZIKV RNA is equally sensitive from serum and plasma. vRNA copies/ml of plasma or serum were quantitated by qRT-PCR over multiple time points from cohort 2 animals. Sufficient baseline samples were not available from serum.

a.

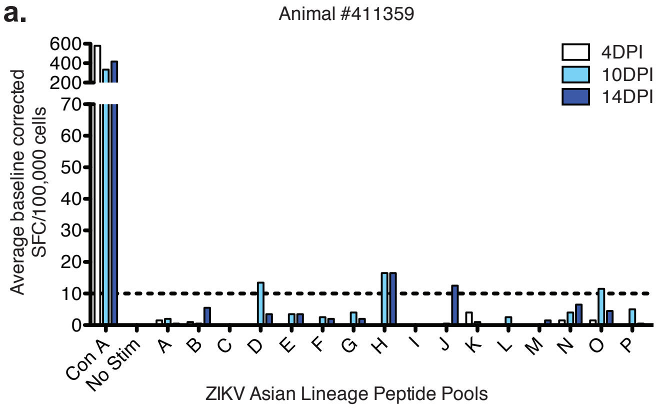

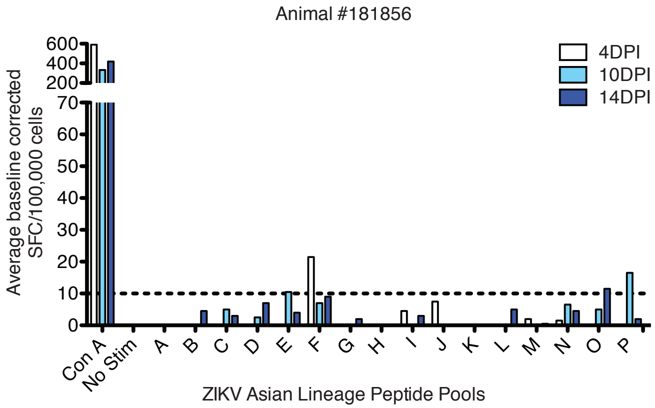

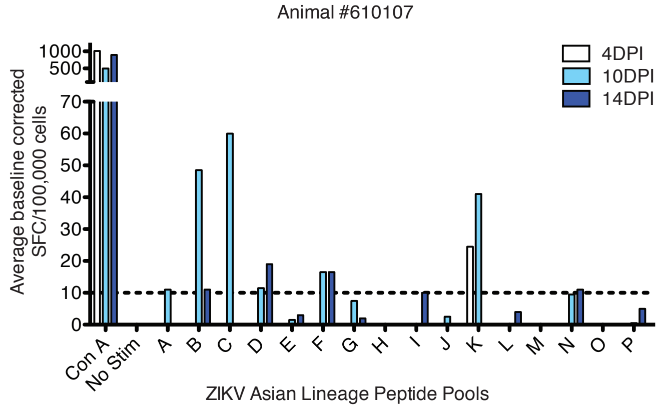

b. Asian lineage ZIKV peptide pools

**Pool ID**	**Target sequence**
A	ZIKV Asian lineage NS5 1-51
B	ZIKV Asian lineage NS5 41-91
C	ZIKV Asian lineage NS5 81-131
D	ZIKV Asian lineage NS5 121-171
E	ZIKV Asian lineage NS5 161-211
F	ZIKV Asian lineage NS5 201-251
G	ZIKV Asian lineage NS5 241-291
H	ZIKV Asian lineage NS5 281-331
I	ZIKV Asian lineage NS5 321-371
J	ZIKV Asian lineage NS5 361-411
K	ZIKV Asian lineage NS5 401-451
L	ZIKV Asian lineage NS5 441-491
M	ZIKV Asian lineage NS5 481-531
N	ZIKV Asian lineage NS5 521-571
O	ZIKV Asian lineage NS5 561-611
P	ZIKV Asian lineage NS5 601-651

c. Summary of NS5-specifc T cell responses

**Animal ID**	**MHC Haplotype**	**DPI**	**Pool ID**	**Target Sequence**
411359	A004, A023, B012b, B024a	10	D	ZIKV Asian NS5 121-171
			**H**	**ZIKV Asian NS5 281-331**
			O	ZIKV Asian NS5 561-575
		14	**H**	**ZIKV Asian NS5 281-331**
			J	ZIKV Asian NS5 361-411
181856	A001, A004, B012b, B055	4	F	ZIKV Asian NS5 201-251
		10	P	ZIKV Asian NS5 601-651
		14	O	ZIKV Asian NS5 561-575
610107	A004, A023, B012b, B048	4	**K**	**ZIKV Asian NS5 401-451**
		10	A	ZIKV Asian NS5 1-51
			**B**	**ZIKV Asian NS5 41-91**
			C	ZIKV Asian NS5 81-131
			**D**	**ZIKV Asian NS5 121-171**
			**F**	**ZIKV Asian NS5 201-251**
			**K**	**ZIKV Asian NS5 401-451**
		14	**B**	**ZIKV Asian NS5 41-91**
			**D**	**ZIKV Asian NS5 121-171**
			**F**	**ZIKV Asian NS5 201-251**
			I	ZIKV Asian NS5 321-371
			N	ZIKV Asian NS5 521-571

Supplementary Figure 3| Antigen-specific T cell responses by IFNγ-ELISPOT. a. Average spot forming cell counts for PBMC collected from each animal at 4, 10 and 14 dpi. Data were baseline corrected by subtracting the average negative control values from each response. A threshold of 10.0 SFC/100,000 cells was set as the minimum value to be considered a positive T cell response, as indicated by the dashed line. **b.** Each pool was comprised of 10 overlapping 15mer peptides offset by 4 amino acids. **c.** Peptide pools eliciting T cell responses at 4, 10 and 14 dpi for each animal. The region of the NS5 protein that is represented by each pool of overlapping 15mers is provided. MHC class I haplotypes of each cohort 2 animal are also presented. All three animals shared the A004 and B012b major histocompatibility complex haplotypes and two animals shared the A023 haplotype. Therefore, it was not surprising that 3 pools were recognized by 2 different animals likely sharing the MHC class I allele that is presenting one of the peptides in those pools. Grayed pools were positive in more than one animal and bolded pools were positive at more than one time point in the same animal.

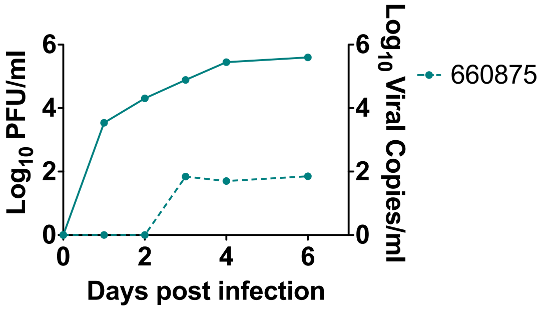

Supplementary Figure 4| Plaque assay titers in a pregnant animal. Log10 PFU/ml serum (dotted line) is plotted relative to vRNA copies/ml plasma (solid line) for 660875.

Supplementary Table 1| Nucleotide variants in ZIKV challenge stock used to infect rhesus macaques

Site relative to KJ776791	Change	Amino Acid Change	Codon Change	Protein Effect	Variant Frequency
801	T -> C	F->L	UUC->CUC	Substitution	17.9%
2318	C -> T		CUC->CUT	None	18.7%
2863	T -> C	L->P	CUC->CCC	Substitution	7.7%
3223	A -> T	K->I	AAA->ATA	Substitution	10.5%
3475	T -> C	M->T	AUG->ACG	Substitution	7.9%
3740	C -> T		GUC->GUT	None	11.1%
3835	C -> T	A->V	GCG->GTG	Substitution	15.8%
4242	G -> C	E->Q	GAG->CAG	Substitution	40.9%

*Only variants found in >5% of sequences are shown.
