# Supplementary material for: A rhesus macaque model of Asian-lineage Zika virus infection
Source: Nat Commun. 2016 Jun 28;7:12204. doi: 10.1038/ncomms12204 (PMC4931337; doi:10.1038/ncomms12204)
Supplement: Peer Review File [file ncomms12204-s2.pdf]

Editorial Note: This manuscript has been previously reviewed at another journal that is not operating a transparent peer review scheme.
